# The association of *HFE* gene H63D polymorphism with endurance athlete status and aerobic capacity: novel findings and a meta-analysis

**DOI:** 10.1007/s00421-020-04306-8

**Published:** 2020-01-22

**Authors:** Ekaterina A. Semenova, Eri Miyamoto-Mikami, Egor B. Akimov, Fatima Al-Khelaifi, Haruka Murakami, Hirofumi Zempo, Elena S. Kostryukova, Nikolay A. Kulemin, Andrey K. Larin, Oleg V. Borisov, Motohiko Miyachi, Daniil V. Popov, Eugenia A. Boulygina, Mizuki Takaragawa, Hiroshi Kumagai, Hisashi Naito, Vladimir P. Pushkarev, Dmitry A. Dyatlov, Eugene V. Lekontsev, Yuliya E. Pushkareva, Liliya B. Andryushchenko, Mohamed A. Elrayess, Edward V. Generozov, Noriyuki Fuku, Ildus I. Ahmetov

**Affiliations:** 1 Department of Molecular Biology and Genetics, Federal Research and Clinical Center of Physical-Chemical Medicine of Federal Medical Biological Agency, Moscow, Russia; 2 Department of Biochemistry, Kazan Federal University, Kazan, Russia; 3 Graduate School of Health and Sports Science, Juntendo University, Chiba, Japan; 4 Central Cross Country Ski Association, Madison, WI USA; 5 Anti Doping Laboratory Qatar, Sports City, Doha, Qatar; 6 UCL-Medical School, Royal Free Campus, London, UK; 7 Department of Physical Activity Research, National Institutes of Biomedical Innovation, Health and Nutrition, NIBIOHN, Tokyo, Japan; 8 Faculty of Health and Nutrition, Tokyo Seiei College, Tokyo, Japan; 9 Institute for Genomic Statistics and Bioinformatics, University Hospital Bonn, Bonn, Germany; 10 Laboratory of Exercise Physiology, Institute for Biomedical Problems of the Russian Academy of Sciences, Moscow, Russia; 11 Omics Technologies OpenLab, Kazan Federal University, Kazan, Russia; 12 Research Fellow of Japanese Society for the Promotion of Science, Tokyo, Japan; 13 Medical Genetic Centre “Progen”, Moscow, Russia; 14 Moscow Center of Advanced Sport Technologies, Moscow, Russia; 15 Department of the Theory of Physical Culture and Biomechanics, Ural State University of Physical Culture, Chelyabinsk, Russia; 16 Methodical and Analytical Department, Regional Center for Sports Training, Chelyabinsk, Russia; 17 Research Institute of Olympic Sports, Ural State University of Physical Culture, Chelyabinsk, Russia; 18 Department of Pediatrics, South Ural State Medical University, Chelyabinsk, Russia; 19 Department of Physical Education, Plekhanov Russian University of Economics, Moscow, Russia; 20 Biomedical Research Center, Qatar University, Doha, Qatar; 21 Laboratory of Molecular Genetics, Kazan State Medical University, Kazan, Russia; 22 Research Institute for Sport and Exercise Sciences, Liverpool John Moores University, Byrom St, Liverpool, L3 5AF UK

**Keywords:** Gene, Genotype, Hemochromatosis, Endurance performance, Athletes

## Abstract

**Purpose:**

Iron is an important component of the oxygen-binding proteins and may be critical to optimal athletic performance. Previous studies have suggested that the G allele of C/G rare variant (rs1799945), which causes H63D amino acid replacement, in the *HFE* is associated with elevated iron indexes and may give some advantage in endurance-oriented sports. The aim of the present study was to investigate the association between the *HFE* H63D polymorphism and elite endurance athlete status in Japanese and Russian populations, aerobic capacity and to perform a meta-analysis using current findings and three previous studies.

**Methods:**

The study involved 315 international-level endurance athletes (255 Russian and 60 Japanese) and 809 healthy controls (405 Russian and 404 Japanese). Genotyping was performed using micro-array analysis or by PCR. VO_2max_ in 46 male Russian endurance athletes was determined using gas analysis system.

**Results:**

The frequency of the iron-increasing CG/GG genotypes was significantly higher in Russian (38.0 vs 24.9%; OR 1.85, *P* = 0.0003) and Japanese (13.3 vs 5.0%; OR 2.95, *P* = 0.011) endurance athletes compared to ethnically matched controls. The meta-analysis using five cohorts (two French, Japanese, Spanish, and Russian; 586 athletes and 1416 controls) showed significant prevalence of the CG/GG genotypes in endurance athletes compared to controls (OR 1.96, 95% CI 1.58–2.45; *P* = 1.7 × 10^–9^). Furthermore, the *HFE* G allele was associated with high V̇O_2max_ in male athletes [CC: 61.8 (6.1), CG/GG: 66.3 (7.8) ml/min/kg; *P* = 0.036].

**Conclusions:**

We have shown that the *HFE* H63D polymorphism is strongly associated with elite endurance athlete status, regardless ethnicities and aerobic capacity in Russian athletes.

## Introduction

Iron is an important component of the oxygen-binding proteins, such as hemoglobin and myoglobin. Whereas hemoglobin transports oxygen (via erythrocytes), myoglobin’s function is to store oxygen in working skeletal muscles and to facilitate its transport to mitochondria. Approximately 65% of iron is stored in hemoglobin (Wallace 2016), thus there is a positive correlation between serum iron concentrations and hemoglobin (Ofojekwu et al. 2013; Baart et al. 2018). Iron can affect many physiological processes, and its deficiency is associated with fatigue, anemia, and decreased exercise performance (DellaValle 2013; Abbaspour et al. 2014). There is a balance between iron loss, iron absorption, and iron storage to maintain iron homeostasis (DellaValle 2013; Wallace 2016; Rubeor et al. 2018). Endurance athletes have an increased risk for iron loss because of the insufficient dietary intake and training intensity, which leads to increased risk for suboptimal iron status (Hinton 2014).

Serum iron measures and hematological parameters have significant heritability components. The heritability estimates are 23% for iron, 29–37% for ferritin, and 28% for transferrin saturation (Njajou et al. 2006; McLaren et al. 2010), and 84% for hemoglobin (Evans et al. 1999). Genetic variation plays a significant role in interindividual differences in serum iron parameters. More specifically, previous studies have suggested that the missense mutations of the hemochromatosis (*HFE*) gene are associated with iron indexes (Burt et al. 1998; Wallace 2016). The proportion of variance explained by *HFE* gene mutations was reported to be 2.1% for serum iron level, 5.6% for ferritin, and 3.5% for transferrin saturation (Njajou et al. 2006).

The *HFE* gene (full name—homeostatic iron regulator) is a protein coding gene located on chromosome 6. The protein regulates iron absorption by regulating the interaction of the transferrin receptor with transferrin. The HFE protein interacts with TFRC, the transferrin receptor, so its primary mode of action is through regulation of the iron storage hormone hepcidin. Individuals with one (C/G or H63D genotype) or two (G/G or D63D genotype) missense mutations of the H63D (also known as His63Asp or rs1799945 C/G) polymorphism, show higher circulating iron concentrations than people without mutations (Burt et al. 1998). In the H63D carrier group, a positive correlation between iron and hemoglobin was noted (Barbara et al. 2016). The H63D mutation is commonly found in European (17%) and American (12%) populations, and is rarer in East Asian (3%), South Asian (7%), and African (1%) populations.

The H63D mutation accounts for a mild form of hereditary hemochromatosis (HH), a condition with increased intestinal iron absorption which may lead to liver fibrosis and cirrhosis, hepatocellular carcinoma, diabetes mellitus, cardiomyopathy, and hypogonadotropic hypogonadism (Wallace 2016). Given the importance of iron and hemoglobin in athletic performance, one might suggest that the *HFE* gene H63D may give some advantage in endurance sports. Indeed, Deugnier et al. (2002) have identified an increased frequency of the G allele in 77 elite French road male cyclists when compared to controls (24.7 vs 17.1%, *P* = 0.04). In accordance with this data, the frequency of the C/G genotype was significantly higher in 65 professional Spanish endurance athletes (50 road cyclists and 15 endurance runners) in comparison with controls (41.5 vs 24.6%, *P* = 0.01) (Chicharro et al. 2004). Finally, Hermine et al. (2015) have found that the frequency of CG/GG genotypes was significantly higher in the group of French elite athletes compared to controls (38% vs 21.9%, *P* = 0.0019). Although these findings in West European populations support the hypothesis that the iron-increasing *HFE* rs1799945 G allele is favorable for endurance performance, replication studies in different ethnic groups using different designs are warranted. This approach leads to the exclusion of false-positive genetic associations (Eynon et al. 2013; Zarebska et al. 2017; Papadimitriou et al. 2018; Yvert et al. 2018; Guilherme et al. 2019; Pickering et al. 2019).

The aim of the study was to investigate the association between the *HFE* gene H63D polymorphism and endurance athlete status in Japanese and Russian populations, aerobic capacity, and to perform a meta-analysis using current findings and three previous studies.

## Methods

### Ethical approval

The study was approved by the Ethics Committee of the Physiological Section of the Russian National Committee for Biological Ethics, Ethics Committees of the Juntendo University and National Institutes of Biomedical Innovation, Health and Nutrition (Japan) and by the Institutional Research Board of Anti-Doping Laboratory Qatar (ADLQ) (F2014000009). Written informed consent was obtained from each participant. The study complied with the guidelines set out in the World Medical Association Declaration of Helsinki and ethical standards in sport and exercise science research. The experimental procedures were conducted in accordance with the set of guiding principles for reporting the results of genetic association studies defined by the Strengthening the reporting of genetic association studies (STREGA) statement.

### Study participants

The study involved 315 international-level endurance athletes (255 Russian and 60 Japanese) and 809 healthy controls (405 Russian and 404 Japanese). The first group comprised 255 international-level Russian endurance athletes tested negative for doping substances and involved in biathlon, kayaking, cross-country skiing, cycling, rowing, running ≥ 800 m, speed skating ≥ 1.5 km, swimming ≥ 400 m, and triathlon. Controls were 405 healthy, unrelated citizens of Russia without any competitive sport experience. Of those, 46 male endurance athletes (middle-distance athletes (*n* = 31): rowers, kayakers, speed skaters; long-distance athletes (*n* = 15): biathletes and cross-country skiers) participated in the study of aerobic performance. The second group involved 60 Japanese international-level endurance athletes (800 m to marathon runners) tested negative for doping substances, including several world record holders and medallist in Olympic Games. Controls were (*n* = 406) healthy, unrelated citizens of Japanese.

### Genotyping

DNA samples of the Russian cohorts were majorly genotyped using micro-array analysis, as described previously (Pickering et al. 2019). In part, some DNA samples of Russian athletes and controls were genotyped for the HFE rs1799945 polymorphism with a TaqMan^®^ SNP Genotyping Assay (Thermo Fisher Scientific Inc, Waltham, Massachusetts, USA) with a StepOne TM Real-Time PCR System (Thermo Fisher Scientific Inc, Waltham, Massachusetts, USA) or using PCR-restriction fragment length polymorphism (RFLP) method, according to a previously described method (Merryweather-Clarke et al. 1997).

Japanese cohort: total DNA was extracted from saliva or venous blood using Oragene DNA Collection Kit (DNA genotek, Ontario, Canada) or QIAamp DNA blood Maxi Kit (QIAGEN, Hilden, Germany), respectively. Illumina^®^ HumanOmniExpress Beadchip (Illumina Inc, Hayward, California, USA) were used for genotyping of more than 700,000 SNPs in athletes and controls. The genotype calls were performed with Illumina^®^ GenomeStudio Software. Genotype data of the *HFE* rs1799945 polymorphism were obtained from the genotyping results of the Illumina^®^ HumanOmniExpress Beadchip.

### VO_2max_ measurement

Maximal oxygen consumption rate (V̇O_2max_) in rowers was determined using an incremental test to exhaustion on a PM 3 rowing ergometer (Concept II, Morrisville, Vermont, USA). The initial workload was 150 W. The duration of exercise at each workload was 3 min, with a 30 s rest period between increments of 50 W. VO_2_ and VCO_2_ was determined breath by breath by a MetaMax 3B gas analysis system (Cortex, Leipzig, Germany) using an electro-chemical cell and non-dispersive infrared sensor, respectively; air flow was measured using a turbine transducer (Triple V). Two-point gas calibrations (first gas—15% O_2_, 5% CO_2_; second gas—ambient air) were performed daily. A one-point gas calibration with ambient air was performed before each test as well as a flow transducer calibration using a 3 L syringe (Hans Rudolph, Kansas City, USA). The criteria used to confirm a maximal test were a decrease in power of more than 30 W from the target power despite strong verbal encouragement and a respiratory exchange ratio greater than 1.1 before cessation of exercise. V̇O_2max_ was recorded as the highest mean value observed over a 30 s period.

V̇O_2max_ in kayakers was determined using an incremental test to exhaustion on a kayaking ergometer (Efremov, Moscow, Russia). The initial workload was 8 kg for men and 5 kg for women. The duration of exercise at each workload was 2 min, with a 30 s rest period between increments of 1 kg. V̇O_2max_ was determined breath by breath using a MetaLyzer II gas analysis system (Cortex Biophysik, Leipzig, Germany). VO_2max_ was recorded as the highest mean value observed over a 30 s period.

V̇O_2max_ in speed skaters was determined using a ramp test to exhaustion on an electromagnetic cycle ergometer Ergoselect 200 K (Ergoline, Bitz, Germany). The initial workload was 60 W, the increment was 15 W/min, and the target cadence was 60–70 rpm. V̇O2max was determined breath by breath using a MetaMax 3B gas analysis system (Cortex Biophysik, Leipzig, Germany). The criteria used to confirm a maximal test were a decrease in cadence of less than 50 rpm despite strong verbal encouragement and a respiratory exchange ratio greater than 1.1 before cessation of exercise. V̇O2max was recorded as the highest mean value observed over a 30 s period.

V̇O_2max_ in biathletes and cross-country skiers was determined using an incremental test to exhaustion on a treadmill HP Cosmos (h/p/cosmos sports & medical gmbh, Nussdorf, Germany). The initial speed was 7 km/h, the increment was 0.1 km/h every 10 s. V̇O_2max_ was determined breath by breath using a MetaMax 3B-R2 gas analysis system (Cortex Biophysik, Leipzig, Germany). V̇O_2max_ was recorded as the highest mean value observed over a 30 s period.

### Selection of studies for the meta-analysis

Databases of PubMed, Web of Science, Science Direct and Google Scholar were searched for association studies as of July 19, 2019. The terms used were “HFE” and “athletes” restricted to English. The exclusion criteria were: (1) review; (2) non-English; (3) studies did not involve endurance athletes; (4) ethnically mixed group of athletes were analysed (given that allelic frequencies vary significantly across different ethnicities; for example, we did not include the study of Grealy et al. (2015) because the mixed group of athletes from North America, Europe, Oceania, South America, Asia, and Africa was studied); and (5) duplicates. The inclusion criteria were: (1) case–control study design evaluating the association between *HFE* gene H63D polymorphism and endurance athlete status; (2) sufficient genotype frequency data to calculate the odds ratios (ORs) and 95% confidence intervals (CIs) and (3) athletes and controls in studies comply with the Hardy–Weinberg equilibrium (HWE). Overall, seven articles published between 1998 and 2015 were identified of which three were found as eligible including a total number of 271 endurance athletes and 607 controls.

### Statistical analysis

Genotype distribution and allele frequencies between athletes and controls were compared using *χ*^2^ tests. Differences in phenotype between groups were analysed using unpaired *t* tests. Data are presented as mean (standard deviation). Statistical analyses were conducted using GraphPad InStat software (GraphPad Software, Inc., California, USA) and PLINK software program (Purcell et al. 2007). To perform the meta-analysis with obtained data and all published studies the Cochrane Review Manager (RevMan) (London, UK) version 5.3 was used. Random and fixed effect models were applied. Odds ratio with 95% confidence intervals (CI) was estimated using the Mantel–Haenszel method. The heterogeneity degree between the studies was assessed with the *I*^2^ statistics. *P* values < 0.05 were considered statistically significant.

## Results

### Case–control study

In Japanese and Russian groups of athletes and controls, the *HFE* gene rs1799945 polymorphism met Hardy–Weinberg expectations (*P* > 0.05 in both groups tested separately). The frequencies of the rs1799945 G allele were significantly higher in Russian (21.0 vs 13.2%; *P* = 0.0002) and Japanese (7.5 vs 2.5%; *P* = 0.0032) endurance athletes compared to ethnically matched controls (Table 1). Furthermore, the rs1799945 CG/GG genotypes were significantly over- represented in Russian (38.0 vs 24.9%; OR 1.85, *P* = 0.0003) and Japanese (13.3 vs 5.0%; OR 2.95, *P* = 0.011) endurance athletes compared to ethnically matched controls (Table 2). These results remained statistically significant after correction for multiple testing.

**Table 1:** Distribution of *HFE* genotypes and allelic frequencies in Japanese and Russian endurance athletes and controls

Groups	*n*	Athletes				*n*	Controls				*P*
		CC	CG	GG	G allele, %		CC	CG	GG	G allele, %	
Russian	255	158	87	10	21.0	405	304	95	6	13.2	0.0002*
Japanese	60	52	7	1	7.5	404	384	20	0	2.5	0.0032*

*P < 0.05, statistically significant differences of G allele frequency between athletes and controls

**Table 2:** Distribution of *HFE* genotypes in endurance athletes and controls

Groups	Athletes			Controls			*P*
	*n*	Genotypes		*n*	Genotypes		
		CC	CG/GG		CC	CG/GG	
Russian	255	158	97 (38.0%)	405	304	101 (24.9%)	0.0003*
Japanese	60	52	8 (13.3%)	404	384	20 (5.0%)	0.011*
French #1 (Deugnier et al. 2002)	77	43	34 (44.2%)	254	173	81 (31.9%)	0.048*
Spanish (Chicharro et al. 2004)	65	36	29 (44.6%)	134	96	38 (28.4%)	0.023*
French #2 (Hermine et. al. 2015)	129	80	49 (38.0%)	219	171	48 (21.9%)	0.0012*

*P < 0.05, statistically significant differences of CG/GG genotypes frequency between athletes and controls

### Meta-analysis

Multi-database literature search yielded three eligible studies involving endurance athletes that were genotyped for the *HFE* gene H63D polymorphism. These involved 77 French elite road cyclists and 254 controls (Deugnier et al. 2002); 65 Spanish highly trained athletes (50 professional road cyclists and 15 Olympic class endurance runners) and 134 controls (sedentary men from Spain) (Chicharro et al. 2004) and 129 French elite athletes (Nordic skiing, rowing, fighting) and 219 controls (Hermine et. al. 2015). The genotypic frequencies for both the cases and the controls in all studies were in Hardy–Weinberg equilibrium.

**Fig. 1:**
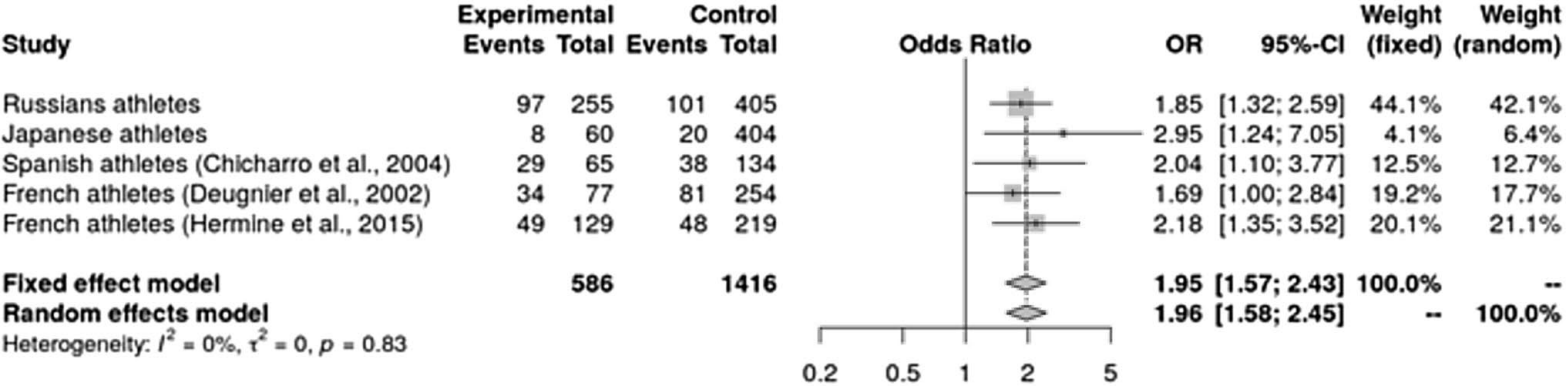
Meta-analysis for association studies for *HFE* gene and endurance sports

The frequencies of the rs1799945 CG/GG genotypes were significantly higher in three groups of French and Spanish endurance athletes compared to controls (Table 2). Overall, five case–control studies (two current and three previous) including a total number of 586 endurance athletes and 1416 controls were used for the meta-analysis. The pooled OR for the CG/GG genotypes compared to the CC genotype was 1.95 (95% CI 1.57–2.43, *P* = 2.5 × 10^–9^ for the fixed effect model) and 1.96 (95% CI 1.58–2.45, *P* = 1.7 × 10^–9^ for the random effect model) (Fig. 1). No heterogeneity between studies (*I*^*2*^ = 0%; *P* = 0.83) was observed. These results indicate that the carriage of the *HFE* mutation (i.e. CG/GG genotypes) is strongly associated with endurance athlete status.

### Aerobic study

We identified that the *HFE* gene rs1799945 G allele was significantly associated with increased V̇O_2max_ in the whole group of Russian male endurance athletes (CC [*n* = 29]: 61.8 (6.1)*,* CG/GG [*n* = 17]: 66.3 (7.8) ml/min/kg; *P* = 0.036), as well as in long-distance athletes only (CC [*n* = 8]: 68.1 (3.4)*,* CG/GG [*n* = 7]: 73.0 (4.6) ml/min/kg; *P* = 0.038).

## Discussion

This is the first study to demonstrate that H63D variation in the *HFE* is associated with elite endurance athlete status in Russian and Japanese populations. More specifically, we found that the frequencies of the iron-increasing genotypes (i.e. CG/GG) were significantly higher in Russian and Japanese elite endurance athletes compared to ethnically matched controls. We also confirmed the observation that the H63D mutation is commonly found in East Europeans (13.2%) and is rarer in East Asian (2.5%) populations. In addition, the meta-analysis using five cohorts (two French, Japanese, Spanish, and Russian) including a total number of 586 endurance athletes and 1416 controls showed significantly higher prevalence of the CG/GG genotypes in endurance athletes compared with controls.

The H63D polymorphism is functional given that the rare G allele has been shown to reduce the ability of the HFE protein to bind to its ligand, thereby preventing the inhibition of transferrin–TFRC binding and resulting in increased transport of iron into circulation and cells (Feder et al. 1998). The hypothesis that the iron-increasing *HFE* rs1799945 G (63D) allele is favorable for endurance performance was confirmed in our functional study, where we identified that the G allele was associated with increased V̇O_2max_ in Russian male endurance athletes. One might suggest that the favorable effect of the *HFE* G allele on aerobic capacity and ability to become an endurance athlete is mediated through its impact on hematological parameters, as was shown in the study of French endurance athletes (Hermine et al. 2015). Furthermore, in the genome-wide association study (GWAS) of 173,480 European-ancestry participants, the *HFE* rs1799945 G allele was shown to be significantly (*P* < 5 × 10^–8^) associated with increased values of hematological parameters, such as hematocrit, mean corpuscular hemoglobin concentration, hemoglobin, and reticulocyte count (Astle et al. 2016). Previous studies in athletes have also shown that variations in genes, which regulate hematological traits, are associated with aerobic capacity and endurance athlete status (Ahmetov et al. 2015; Malczewska-Lenczowska et al. 2016).

Our findings seem reasonable given the importance of iron metabolism for endurance athletes (Abbaspour et al. 2014). The leading role of iron is to transport oxygen into the red blood cells and tissues, and it does so mainly through hemoglobin. Furthermore, iron is present in myoglobin and cytochromes of skeletal muscle mostly in oxidative (slow-twitch) muscle fibers. The normal level of iron is crucial to maintain redox balance in muscle and produce mitochondrial energy production, which are primary factors determining exercise performance (Buratti et al. 2015). Iron deficiency without anemia and/or sports anemia are a common issue in athletic populations (at 15–35% of female and 3–11% of male athletes) (Fallon 2008; Malczewska et al. 2001; Parks et al. 2017) with higher frequency in endurance athletes, e.g., distance runners and triathletes (Rietjens et al. 2002; Lukaski 2004; Sinclair and Hinton 2005), and physically active individuals compared with sedentary controls (Milic et al. 2011; Eliakim et al. 2002; Gropper et al. 2006; Woolf et al. 2009). Factors, which could affect both male and female athletes’ iron stores, is low energy intake, inadequate dietary iron intake, vegetarian diets, exercise-associated iron losses, reduced iron recycling (Hinton 2014; Castell et al. 2019; Sim et al. 2019), and menstrual blood losses in female athletes (Pedlar et al. 2018). Iron deficiency, which is accompanied by a reduction of oxygen transport to the working skeletal muscle, can lead to lower blood pH, depletion of muscle glycogen, which may negatively affect the endurance performance and exercise economy (Sim et al. 2019).

Humans with experimentally induced anemia showed reduced VO_2max_, which is proportional to hemoglobin concentrations (Woodson et al. 1978; Celsing et al. 1986). Iron supplementation of anemic women improved iron status and performance during a standardized, multi-stage treadmill test and reduced exercise heart rate and blood lactate concentrations (Gardner et al. 1975). Although iron supplementation does not necessarily improve VO_2max_ (Klingshirn et al. 1992; Zhu and Haas 1998), this strategy is useful for iron-deficient nonanemic athletes in the improvement of athletic performance in endurance sports (Burden et al. 2015; Rubeor et al. 2018). Therefore, the iron status of athletes should be monitored systematically throughout the training and competition season to early detection or prevention of iron deficiency.

The limitation of our study is the small sample sizes of Japanese athletes, as well as sub-group of Russian athletes with VO_2max_ data, which may lead to potential type I errors. As in all such studies, extension to, and replication within other racial groups is proposed.

In conclusion, we have shown that the *HFE* gene H63D polymorphism is strongly associated with endurance athlete status across East Asian, East and West European populations and with aerobic capacity in Russian athletes.

## Abbreviations

CI: Confidence intervals
DNA: Deoxyribonucleic acid
EDTA: Ethylenediaminetetraacetic acid
GWAS: Genome-wide association studies
HFE: Homeostatic iron regulator (hemochromatosis gene)
HH: Hereditary hemochromatosis
HWE: Hardy–Weinberg equilibrium
PCR: Polymerase chain reaction
RFLP: Restriction fragment length polymorphism
SNP: Single-nucleotide polymorphism
STREGA: Strengthening the reporting of genetic association
TFRC: Transferrin receptor
V̇O_2max_: Maximal oxygen consumption
